# Reproducible Capillary Electrophoresis–Mass Spectrometry–Based Top‐Down Proteomics of Complex Proteomes Enabled by an Advanced Cationic Polymer Coating

**DOI:** 10.1002/jms.70005

**Published:** 2025-12-26

**Authors:** Guangyao Gao, Fei Fang, Yifan Yue, Alex T. Zhang, Qianjie Wang, Qianyi Wang, Guijie Zhu, Liangliang Sun

**Affiliations:** ^1^ Department of Chemistry Michigan State University East Lansing Michigan USA

**Keywords:** cationic polymer coating, CZE‐MS, proteoform, reproducibility, top‐down proteomics

## Abstract

Capillary zone electrophoresis–mass spectrometry (CZE‐MS) has become a powerful tool for top‐down proteomics (TDP). Capillaries are commonly coated with neutral or cationic polymers to reduce protein adsorption in CZE‐MS. We recently introduced a cationic polymer coating [poly(acrylamide‐co‐(3‐acrylamidopropyl) trimethylammonium chloride (PAMAPTAC)] to CZE‐MS–based TDP. Here, we further improved the coating procedure to enhance the ease and repeatability of the cationic coating preparation by employing 2,2′‐azobisisobutyronitrile (AIBN) as the radical initiator and methanol as the solvent. The new coating method not only simplifies the coating process but is also user‐friendly. Different laboratory members were able to reproduce this type of cationic coating with consistent CZE‐MS performance. We evaluated the performance of CZE‐MS with the cationic coating in analyzing various complex biological samples (nanoparticle protein corona, histone, and an *Escherichia coli* cell lysate) and assessed its long‐term reproducibility with an *E. coli* cell lysate for 35 runs (over 40 h) of continuous measurement. The CZE‐MS system produced reproducible measurements for all the samples regarding separation profiles, proteoform migration time (MT), and proteoform intensity. For example, the system enabled the detection of large proteoforms (26–35 kDa) from an *E. coli* sample with consistent MT, relative standard deviations (RSDs) <7% without MT correction and 2.1% after MT alignment. We expect that the APTAC coating will further advance CZE‐MS–based TDP for broad and large‐scale applications.

## 1 Introduction

Mass spectrometry (MS)–based top‐down proteomics (TDP) is now a powerful method across diverse studies for quantifying and identifying proteoforms in biological contexts [1, 2, 3], including but not limited to cancer [4, 5, 6, 7], infectious disease [8, 9], and immunology [10, 11]. In TDP studies, capillary zone electrophoresis–mass spectrometry (CZE‐MS) has become a valuable technique providing efficient separations of proteoforms according to their charge‐to‐size ratios and high sensitivity for proteoform detection [12, 13, 14, 15, 16].

Protein adsorption to the capillary inner wall is a major factor limiting the resolution and reproducibility of CZE‐MS. To minimize nonspecific protein adsorption during separation, the inner wall of the capillary is often coated with neutral polymers, e.g., linear polyacrylamide (LPA) [12, 13]. Besides the neutral coatings, the cationic polymer coatings have also been used in ce‐MS–based TDP studies [14, 15, 16, 17]. Using a cationic (positively charged) polymer creates a positively charged layer on the capillary's inner surface [18]. This layer repels proteoforms, which are typically positively charged under typical ce‐MS–based TDP conditions. This reduces adsorption and enhances separation [19]. Cationic capillary coatings can be further categorized into dynamic, semipermanent, and permanent types, depending on whether the coating material is covalently bonded to the silanol group on the capillary inner wall [20]. Although a dynamic cationic coating creates a removable layer and is easy to implement, permanent positively charged coatings have been used for proteoforms by several research groups in many studies [16, 21, 22, 23, 24, 25]. The PEI (polyethyleneimine) coating is one well‐recognized cationic coating for CZE‐MS–based proteomics research [16, 26, 27, 28]. Recently, the poly(acrylamide‐co‐(3‐acrylamidopropyl) trimethylammonium chloride (PAMAPTAC) coating was evaluated for CZE separation of intact proteins by the Václav Kašička group [29], whereas the CZE‐MS–based TDP of complex samples was evaluated by our group [17]. The CZE‐MS–based TDP data from our group demonstrated the value of the PAMAPTAC coating for TDP of large proteoforms in complex biological samples. In this work, we further improved the cationic coating procedure and evaluated the long‐term reproducibility of the cationic coating–based CZE‐MS for measuring proteoforms in various biological systems. The results demonstrate reproducible measurement of proteoforms in various complex samples, and the cationic coating could be made reproducibly by different students in our laboratory. This work laid the foundation for employing the cationic coating for CZE‐MS–based TDP of complex samples.

## 2 Experimental

### 2.1 Materials and Chemicals

The silica capillaries (50 μm i.d., 360 μm o.d.) were purchased from Polymicro Technologies (Phoenix, AZ). Amicon Ultra centrifugal filter units (0.5 mL, 10 kDa cutoff) were purchased from Sigma‐Aldrich (St. Louis, MO). Histone extract from calf thymus was purchased from Roche (product no. 10223565001). Polymerization initiator, 2,2′‐azobis(2‐methylpropionitrile) (AIBN), and monomer (3‐acrylamidopropyl) trimethylammonium chloride (APTAC), 45% hydrofluoric acid were also purchased from Sigma‐Aldrich (St. Louis, MO). LC/MS grade methanol, water, acetic acid, and formic acid were purchased from Fisher Scientific (Pittsburgh, PA). Acrylamide was purchased from Acros Organics (NJ, USA). Additional reagents include protease inhibitor (cOmplete ULTRA Tables, Roche) and urea (Alfa Aesar).

### 2.2 Sample Preparation and Buffer Exchange

A standard protein mixture (0.7 mg/mL bovine serum albumin, 0.3 mg/mL carbonic anhydrase, 0.2 mg/mL myoglobin, and 0.05 mg/mL ubiquitin) was prepared in the background electrolyte (BGE) solution consisting of 5% acetic acid (pH 2.4). The histone was dissolved in water to prepare a concentration of 2 mg/mL histone samples for CZE‐MS analysis.

The protein corona was formed on the BioMagPlus Amine modified magnetic particles (Bangs Laboratories Inc., Fishers, IN) according to the procedure [30] with minor modifications. The magnetic particles (50 mg/mL, 40 μL) were incubated with 55% human plasma (300 μL) for 1 h at 37°C with constant stirring to form protein coronas. The magnetic rack was applied to remove unbound and plasma proteins that were only loosely attached to the surface of magnetic particles. The collected magnetic particles were washed three times with cold DPBS. Then, the protein corona–coated magnetic particles were incubated in a 1% (w/v) SDS solution for 3 h at 60°C with constant agitation. After separating the magnetic particles by magnetic rack, the collected protein corona sample was cleaned through a buffer exchange step with an Amicon Ultra Centrifugal Filter with a molecular weight cutoff (MWCO) of 10 kDa. A total of 200 μg of protein was added to each of the prewetted filters. Then, 200 μL of 50 mM ammonium bicarbonate (ABC), water, and 5% acetic acid was added in sequence and washed via centrifugation at 12°C,14 000×*g* for 30 min. The protein corona samples were washed with ABC three times, with water for twice, and with 5% acetic acid once. The resulting solution in 5% acetic acid was then ready for CZE‐MS analysis.

The *Escherichia coli* sample (strain MG1655) was cultured according to the previously reported procedure [3], and the pellet (0.35 g) was dissolved in 2 mL of the extraction buffer (8 M urea, 100 mM ammonium bicarbonate; cOmplete protease inhibitor). The sample was then sonicated for 2 min twice in an ice bath with a Branson Sonifier 250 (VWR Scientific). The concentration of the collected supernatant was determined using a bicinchoninic acid (BCA) kit. A total of 60 μL of *E. coli* sample lysate solution was added to each of the eight prewetted 10 kDa cutoff Amicon ultra vertical filters. After centrifugation, the proteins on the membrane were washed with three different buffers successively via centrifugation at 14 000×*g* for 35 min at 15°C: 200 μL of the 50 mM ABC solution (pH 8.2), water, and 5% (v/v) acetic acid (pH 2.4). The proteins were washed with each buffer twice. The protein solution, after cleanup on the membrane filters, was collected from the eight filters and combined into one 1.5 mL Eppendorf tube for future experiments.

### 2.3 Capillary Preparation

Bare fused silica capillaries (1 m in length, 50 μm i.d., 360 μm o.d.) were flushed sequentially with 150 μL of 1 M hydrochloric acid (HCl), water, 1 M sodium hydroxide (NaOH), water, and methanol. The capillaries were then dried under nitrogen gas (20 psi) for 2 h. Subsequently, 50% (v/v) 3‐(trimethoxysilyl) propyl methacrylate in methanol was injected into the capillary using a 1 mL plastic syringe. The capillaries were then sealed with rubber stoppers and incubated at room temperature for 1 day. After incubation, the capillaries were flushed with methanol and dried under nitrogen overnight.

The monomer solution, 500 μL of APTAC solution (1.4 mol/L) in methanol was prepared. A total of 20 μL of 5% (w/v) AIBN solution in methanol was added to the cationic monomer solution to initiate the reaction. The mixture was degassed under nitrogen at 30 psi for 10 min. The pretreated capillaries were then filled with the degassed solution using a vacuum pump. The capillaries were then sealed with rubber stoppers and incubated at 40°C for 1 h. The capillaries were then flushed with water to remove any excess monomer solution. To reduce the outer diameter of the coated capillary for CZE‐MS, one end of the capillary was etched by 45% hydrofluoric acid (HF) for 70 min based on the procedure in reference [31].

### 2.4 CZE‐MS

The CZE experiments were performed using an A 7100 ce System from Agilent Technologies (Santa Clara, CA). The CZE system was coupled to a 6545XT AdvanceBio Q‐TOF mass spectrometer (Agilent Technologies, Santa Clara, CA) via an electrokinetically pumped sheath‐flow nanospray interface (EMASS‐II ce‐MS Ion Source, CMP Scientific, Brooklyn, NY) [31, 32, 33]. The sheath buffer consisted of 10% (v/v) methanol and 0.2% (v/v) formic acid in water. The electrospray ionization (ESI) emitters for the CZE‐MS interface were made by pulling borosilicate glass capillaries (1.0 mm outer diameter, 0.75 mm inner diameter, 10 cm length) using a Sutter P‐1000 Flaming/Brown micropipette puller. The final emitter opening size was 30–40 μm. The applied ESI voltage ranged between +2.2 and +2.5 kV, and the distance between the emitter tip and the ion transfer tube was set at 3 mm.

CZE separations were performed by applying a −30 kV voltage at the sample injection end and a 2.2–2.5 kV voltage at the ce‐MS interface. A 5% (v/v) acetic acid solution (pH 2.4) was used as both BGE and sample buffer. The sample junction volume was approximately 50 nL for each run. After each run, the capillary was flushed and cleaned with BGE for 20 min at 20 psi pressure with the application of a −30 kV voltage at the sample injection end.

The gas temperature was 320°C, and the flow rate of the nitrogen drying gas was 1 L/min. The sheath gas temperature and gas flow were 60°C and 2 L/min. The voltage applied to the ion transfer tube was set to 0 V. The mass range of detection was from 600 to 2500 *m*/*z*. The acquisition rate was 0.4 spectra per second.

Data analysis and visualization were performed using R version 4.4.1. Base R packages were used along with additional packages: ggplot2 [34], scales [35], ggrepel [36], patchwork [37], dplyr [38], tidyr [39], and readxl [40].

## 3 Results and Discussion

**FIGURE 1:**
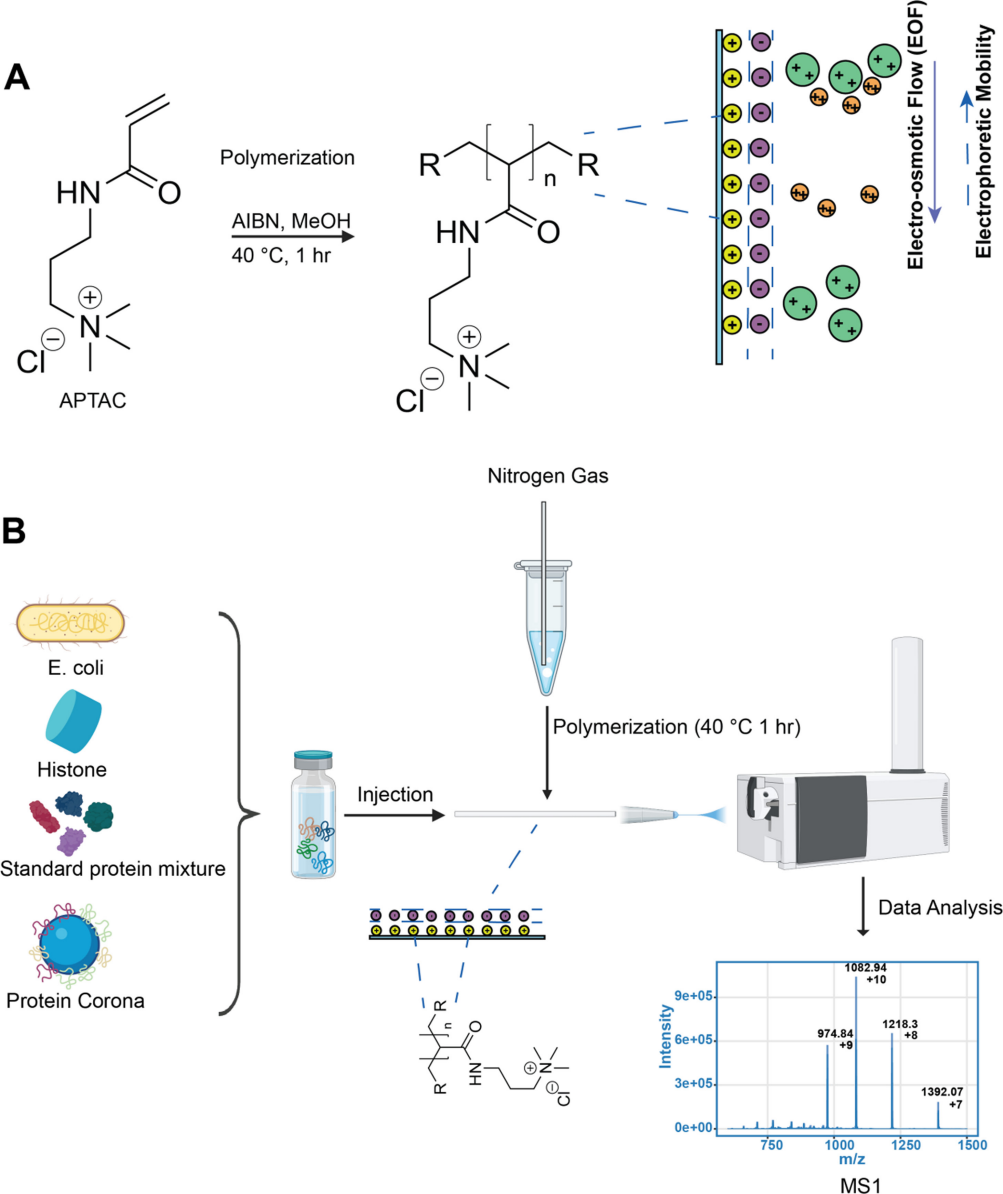
(A) Illustration of a cationic‐coated capillary using APTAC monomer. (B) The schematic diagram of this study (created using BioRender and used here with permission).

In our previous work, we developed a novel CZE‐MS–based TDP technique based on PAMAPTAC coating and preliminarily evaluated its performance for TDP of a complex proteome sample [17]. In that procedure, we employed acrylamide and APTAC as dual monomers and ammonium persulfate (APS) as the polymerization initiator. The reaction was performed in an aqueous condition (i.e., deionized water). The coated capillary showed promising data in TDP applications. However, we realized that it is a little bit difficult for different laboratory members to achieve reproducible data using that procedure. We speculated that the phenomenon was related to the positive charge of monomer APTAC in the aqueous condition during the polymerization process. The positive charge on APTAC molecules attached to the capillary inner wall impedes the movement of additional APTAC molecules toward the capillary inner wall for polymerization due to the electrostatic repulsion, which makes the coating preparation sensitive. In this work, we first aim to improve the repeatability of the coating procedure across different students in the laboratory by switching to a methanol solvent, only one monomer (APTAC), and 2,2′‐azobisisobutyronitrile (AIBN) as the radical initiator, considering the solubility in methanol. We expect that the methanol solvent will facilitate the polymerization because APTAC will have much less dissociation in methanol compared to in water. In this work, we investigated the reproducibility of the new coating procedure across three laboratory members using a standard protein mixture, and more importantly, evaluated the reproducibility of the cationic coating–based CZE‐MS measurement of multiple complex samples (an *E. coli* cell lysate, a histone sample, and one nanoparticle protein corona sample) to make sure the system is broadly applicable. The experimental design is shown in Figure 1.

### 3.1 Repeatability of the Cationic Coating Across Multiple Laboratory Members for CZE‐MS Analysis of Intact Proteins

**FIGURE 2:**
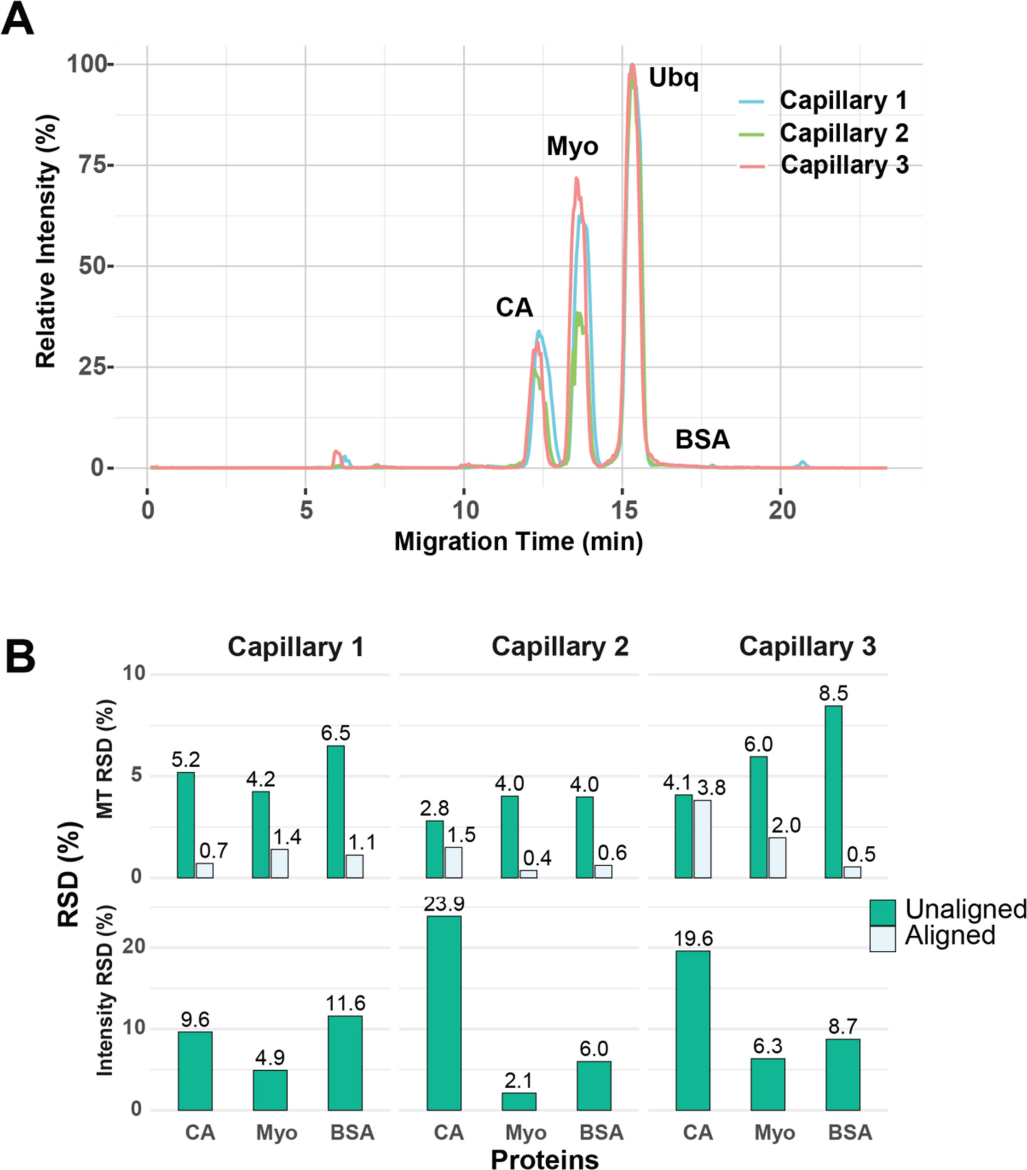
(A) Electropherograms of three coated capillaries prepared by three different laboratory members with alignment. (B) Relative standard deviations (RSDs) for migration time (MT) with/without alignment using base peak and RSDs for intensity.

Three laboratory members prepared the cationic coating using the new procedure. We need to highlight that one of the three laboratory members was a local high school student in his senior year (Alex Zhang). We evaluated the three separation capillaries prepared by different laboratory members using a standard protein mixture containing carbonic anhydrase (CA), myoglobin (Myo), ubiquitin (Ubq), and bovine serum albumin (BSA). Triplicate analyses were performed for each capillary. As shown in Figure 2A, the separation profiles of the standard proteins across the three laboratory members were consistent. In addition, the three separation capillaries showed consistent migration times across triplicate analyses and across the three capillaries, Figure 2B. Without migration time (MT) alignment, the relative standard deviations (RSDs) of MT of proteins are less than 9% across triplicate analysis and less than 8% across capillaries. After a simple MT alignment using the ubiquitin peak as the reference, the RSDs were reduced to less than 2% for triplicate analysis and less than 2% for three capillaries. In terms of proteoform intensity, the RSDs ranged from 2% to 24% across the triplicate analyses and were smaller than 18% across the three capillaries. The highly reproducible MT of proteoforms after a simple alignment and reasonably consistent proteoform intensity suggest that the cationic coating can be prepared with high repeatability for CZE‐MS–based intact proteoforms. We need to emphasize that the possibility of making a reliable capillary coating by a senior high school student after some brief training further makes this technique easy for broad adoption.

### 3.2 Evaluating the Cationic Coating–Based CZE‐MS for Complex Proteoform Mixtures

**FIGURE 3:**
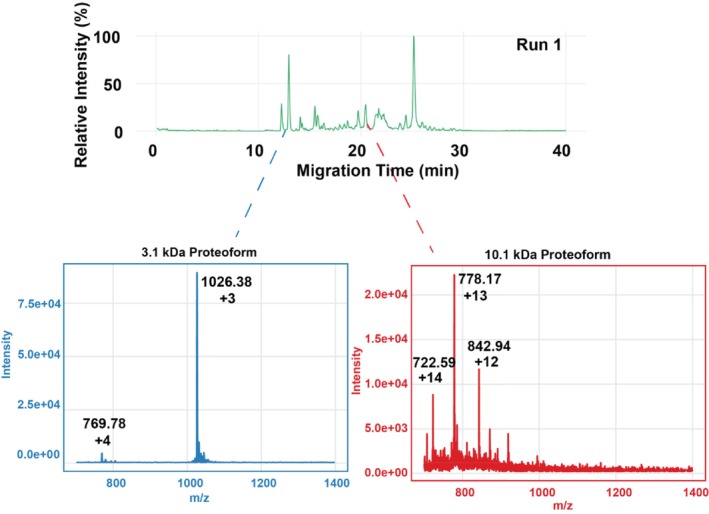
An example electropherogram of a protein corona sample by CZE‐MS. Mass spectra of two example proteoforms are shown with the deconvoluted masses labelled.

**FIGURE 4:**
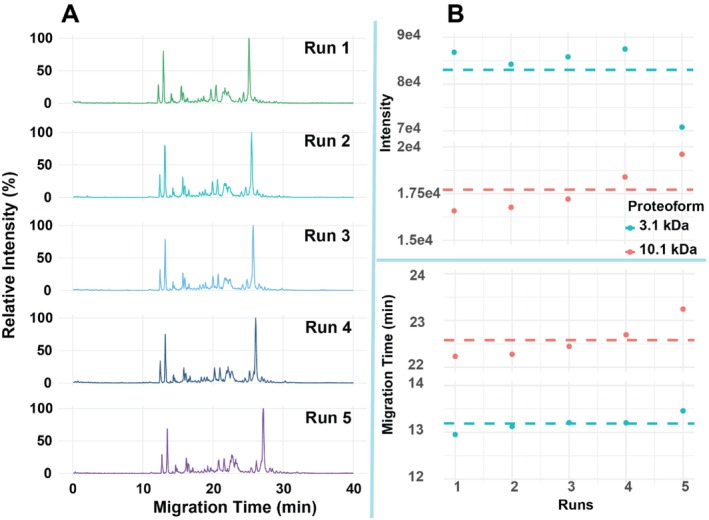
(A) Electropherograms of a protein corona sample after CZE‐MS analysis. (B) Scatter plot of migration time (MT) and signal intensity of two example proteoforms across the five CZE‐MS runs.

We further evaluated three different biological samples to further assess the robustness of the CZE‐MS system. Firstly, we performed five replicate analyses of a protein corona sample [30, 41, 42]. The sample was prepared according to our previous work [30, 41, 42, 43, 44, 45], and a brief procedure was described in the Methods section. The CZE‐MS produced efficient separations of the protein corona proteoforms with an approximately 20‐min separation window. As shown in Figure 3, two example proteoforms with molecular weights (MW) of ~3.1 and 10.1 kDa were clearly detected. The CZE‐MS technique produced reproducible separation and measurement of the protein corona sample across five runs regarding separation profiles (Figure 4A), proteoform intensity, and migration time (Figure 4B). With the two selected proteoforms, both MT and signal intensity were recorded. The 3.1 kDa proteoform exhibited an average MT of 13.19 min with a low standard deviation (SD) of 0.18 min and RSD of 1.39%, while the 10.1 kDa proteoform had a MT of 22.58 min with 0.41 min of SD and 1.83% RSD. Although signal intensities are naturally more prone to fluctuate, especially with ESI, the experimental results remain reproducible. The 3.1 kDa proteoform had an average intensity of around 83 000 with an RSD of 8.36% and the 10.1 kDa proteoform showed around 17 700 signal intensity with 7.17% RSD. These results suggest the consistency and reproducibility of the APTAC‐coated capillary‐based CZE‐MS for analysis of protein corona samples, highlighting the high potential of the CZE‐MS technique for large‐scale TDP analysis of protein coronas.

**FIGURE 5:**
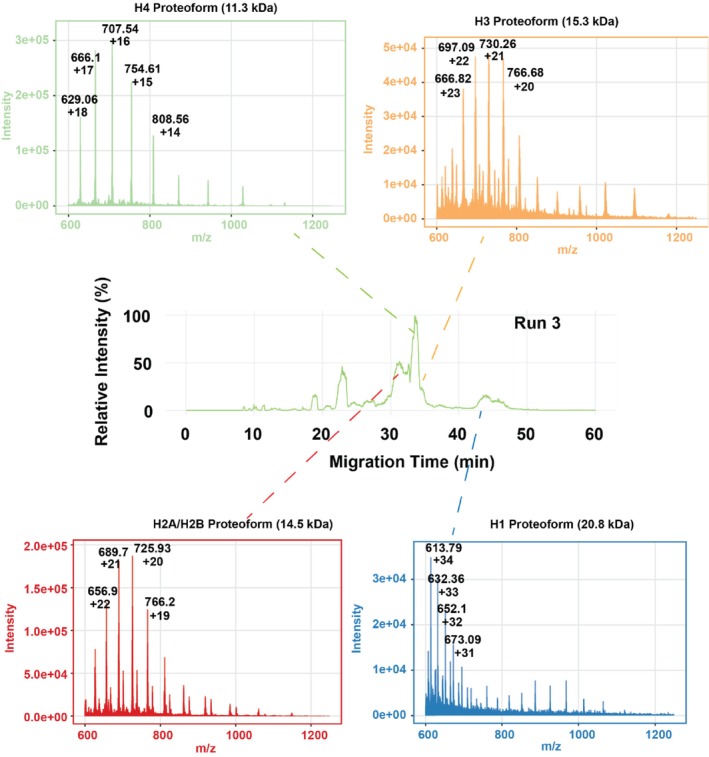
An example electropherogram of the commercial histone sample with mass spectra of the main histone proteins labelled.

**FIGURE 6:**
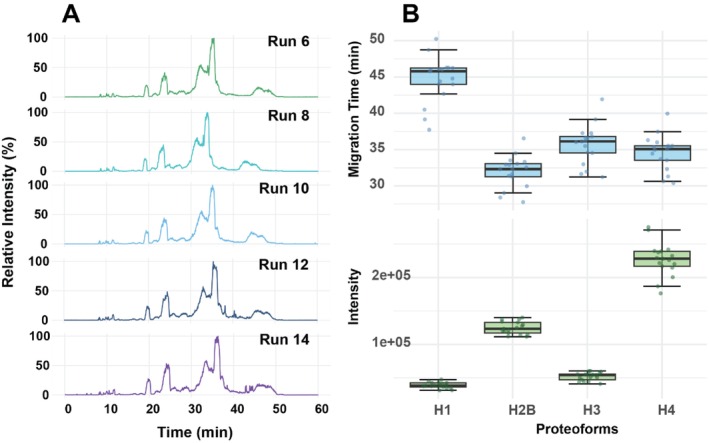
(A) Selected electropherograms of the commercial histone sample after CZE‐MS analysis. (B) Boxplots for migration time (MT) and proteoform intensity distributions across all the CZE‐MS runs for all histone proteins.

Secondly, we further evaluated the CZE‐MS technique using a commercial histone sample. We have performed CZE‐MS–based TDP of histone samples in several studies using LPA‐coated capillaries [7, 46, 47, 48]. The migration order of histone proteoforms using the APTAC coating was reversed compared to the LPA coating [48] as we expected, because of the reversed electroosmotic flow (EOF) in the capillary under the APTAC coating condition Figure 5. One example electropherogram with mass spectra of different histone proteins is also shown in Figure 5, indicating clear separation and measurement of different histone proteoforms Figure 5. In our system, H2B/H2A experienced the shortest MT, followed by H4, H3, and H1, which experienced the longest migration time. In contrast, LPA coating yields the opposite order of H1, H3, H4, and H2A/H2B [48]. One advantage of the cationic‐coated capillary compared to the LPA‐coated capillary is a wider separation window of histone proteoforms. LPA coating resulted in a short separation window of around 6 min [48], whereas the cationic capillary achieved a separation window of about 12 min. A longer separation window allows for a better resolution between closely migrating proteoforms and more time for tandem mass spectrometry (MS/MS) analysis, improving the number of histone proteoform identifications and the performance for label‐free quantification. We performed 18 CZE‐MS runs of the histone sample, and the electropherograms are shown in Figure S1. Electropherograms of five selected CZE‐MS runs are shown in Figure 6A. Our CZE‐MS system generated highly reproducible separation profiles of the histone sample across 18 runs. The migration times and proteoform intensities are also consistent across the runs for all the histone proteins evidenced by the relatively tight distributions (Figure 6B), with RSDs less than 10% and 12% for migration time and intensity, respectively. These results demonstrate that our APTAC coating–based CZE‐MS is robust and consistent for TDP analysis of histone proteoforms.

**FIGURE 7:**
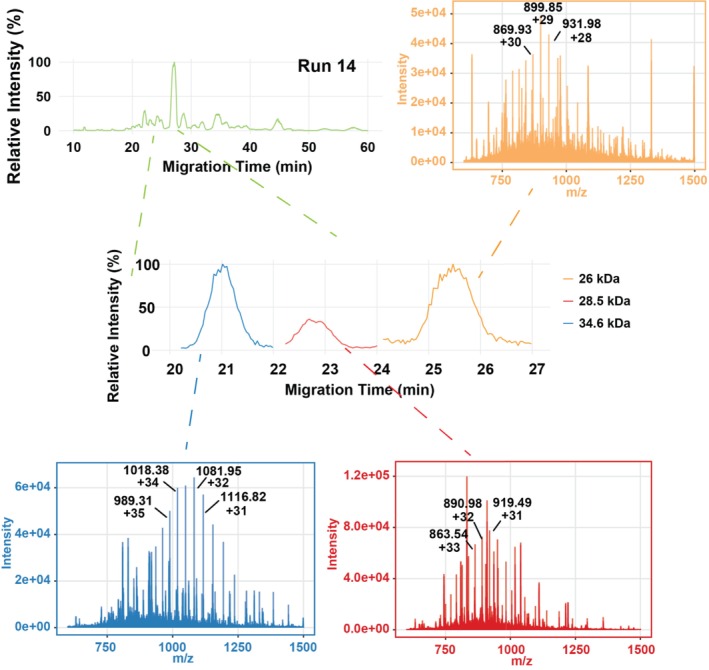
An electropherogram of an *E. coli* cell lysate by CZE‐MS. The extracted ion electropherograms and mass spectra of three selected large proteins (26, 28.5, and 34.6 kDa) are shown.

**FIGURE 8:**
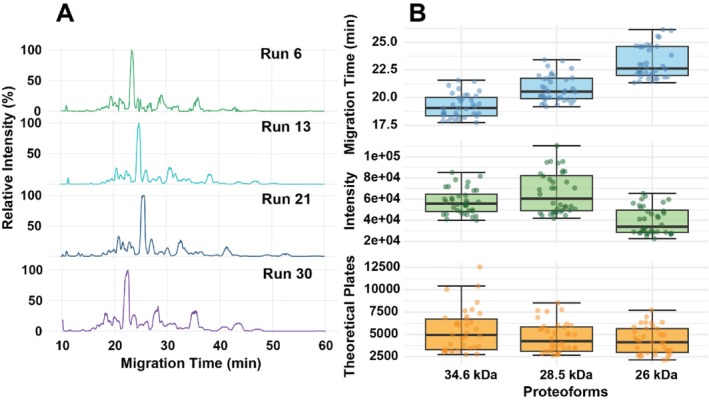
Summary of the *E. coli* sample data from our cationic coating–based CZE‐MS. (A) Selected electropherograms of four CZE‐MS runs. (B) Boxplots of migration time, signal intensity, and the number of theoretical plates of three selected large proteoforms across all the CZE‐MS runs.

Lastly, we tested the CZE‐MS method using an *E. coli* cell lysate with much higher complexity than the protein corona and histone samples. Building on the previous studies on protein corona and histone samples, here we focused on the long‐term reproducibility of the CZE‐MS system. In this study, 35 consecutive runs of an *E. coli* sample were performed with nearly 40 h of MS time, and three relatively large proteoforms ranging from 26 to 34.6 kDa were selected for detailed evaluation. Figure 7 shows an example of the electropherogram of the *E. coli* lysate, with a zoomed‐in view of the selected large proteoforms around 20–27 min (34.6, 28.5, and 26 kDa). The corresponding mass spectra are also displayed with their MTs indicated on the extracted ion chromatogram (EIC). The CZE‐MS enabled the detection of large proteoforms with clear signals. Figure 8A presents electropherograms from several selected runs. The electropherograms of all 35 CZE‐MS runs are shown in Figure S2. The proteoform profile is consistent across the 35 runs, suggesting excellent long‐term reproducibility of our CZE‐MS system for measuring a complex proteoform sample. We further plotted the distributions of MT, signal intensity, and the number of theoretical plates (N) of the three selected large proteoforms across the 35 runs Figure 8B. The tight distributions of MTs for the three proteoforms showed optimal reproducibility over the 35 consecutive runs, the RSDs smaller than 6.5% without MT alignment and less than 2.1% after MT alignment based on the most abundant peak, confirming the reproducible measurement of proteoforms (i.e., large ones) in complex samples by CZE‐MS. For proteoform intensity, the RSD values ranged from 20.9% to 32.8% for the three large proteoforms, indicating reasonable quantitative reproducibility of the method for large‐scale proteoform studies, even for large proteoforms. The relatively larger RSDs of intensity for large proteoforms are most likely due to their low signal intensity (E4) resulting from broad charge state distributions. In terms of the number of theoretical plates (*N*), the data are consistent with the proteoform intensity. The average number of theoretical plates is about 5000 for those large proteoforms. The *N* is much smaller than our data from LPA‐coated capillaries [49, 50], which is due to the lack of online sample stacking in our cationic coating–based CZE‐MS analysis.

## 4 Conclusions

In this study, we developed an improved procedure for making APTAC‐based cationic capillary coating for CZE‐MS–based TDP applications. The improved procedure demonstrated excellent repeatability by different laboratory members, including one senior high school student. The CZE‐MS technique showed highly reproducible measurement of proteoforms in various complex biological systems, including protein corona, histone, and *E. coli* cell lysate samples. The technique also demonstrated its capability for large‐scale TDP studies of complex proteoform samples with reproducible proteoform migration times and intensities across 35 CZE‐MS runs. The results clearly documented the high potential of our new CZE‐MS platform with the cationic coating for large‐scale TDP applications.

Some further improvements are needed to boost its performance for the TDP of complex samples. First, in this study, we did not perform online sample stacking during CZE‐MS analysis, which impeded the system's capability for large‐volume sample loading for detecting more low‐abundance proteoforms. We will investigate the sample stacking techniques for the cationic coating–based CZE‐MS in our future studies. Second, we need to explore the possibility of broad adoption of the technique by different research groups conducting ce‐MS–based protein analysis. We will include the cationic coating in our annual ce‐MS summer school at my research group at Michigan State University to facilitate the broad adoption of the technique.

## Funding

This work was supported by the National Science Foundation (DBI1846913), the National Institute of General Medical Sciences (R35GM153479), and the National Cancer Institute (R01CA247863).

## Conflicts of Interest

The authors declare no conflicts of interest.

## Supporting information


**Figure S1:** Electropherograms for all histone runs (A–C).
**Figure S2:** Electropherograms for all E. coli runs (A–G).
