# Supplementary material for: Reproducible Capillary Electrophoresis–Mass Spectrometry–Based Top‐Down Proteomics of Complex Proteomes Enabled by an Advanced Cationic Polymer Coating
Source: J Mass Spectrom. 2025 Dec 26;61(1):e70005. doi: 10.1002/jms.70005 (PMC12742992; doi:10.1002/jms.70005)
Supplement: Supplementary file 1 — Figure S1: Electropherograms for all histone runs (A–C). Figure S2: Electropherograms for all E. coli runs (A–G). [file JMS-61-e70005-s001.docx]

**Supporting Information**

**Reproducible capillary electrophoresis-mass spectrometry-based top-down proteomics of complex proteomes enabled by an advanced cationic polymer coating**

Guangyao Gao, Fei Fang, Yifan Yue, Alex T. Zhang, Qianjie Wang, Qianyi Wang, Guijie Zhu*, Liangliang Sun*

Department of Chemistry, Michigan State University, East Lansing, Michigan 48824, United States

* Corresponding author.

Guijie Zhu, Email: zhuguiji@msu.edu

Liangliang Sun, Email: [lsun@chemistry.msu.edu](mailto:lsun@chemistry.msu.edu)

Phone: 1-517-353-0498

**Content Page**

Figure S1: Electropherograms for Histones. 2

Figure S2: Electropherograms for E. coli. 3-6


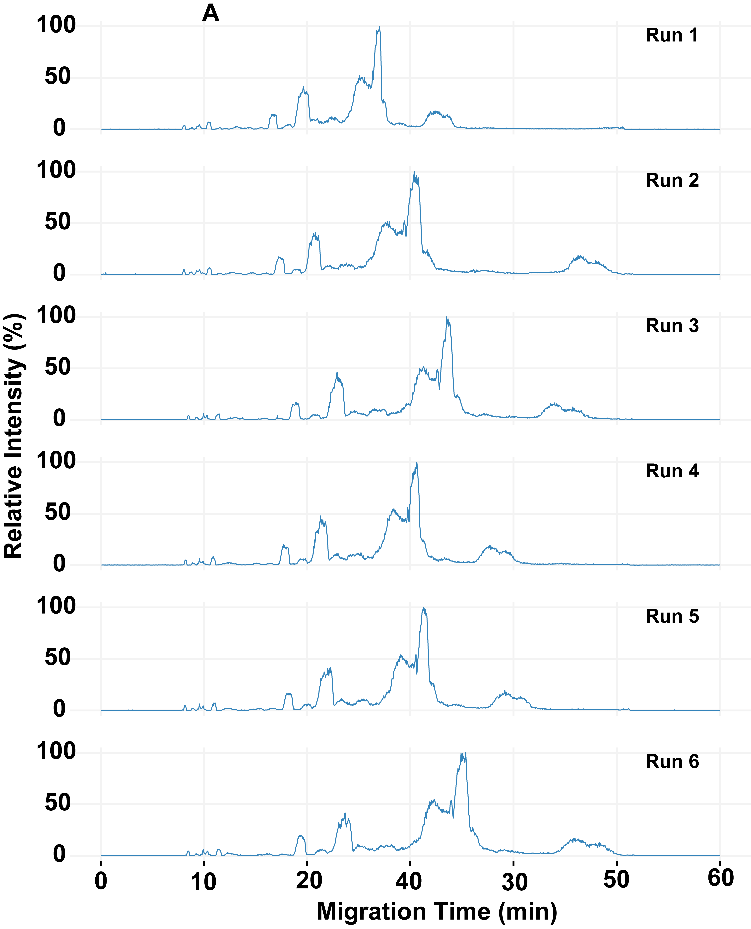

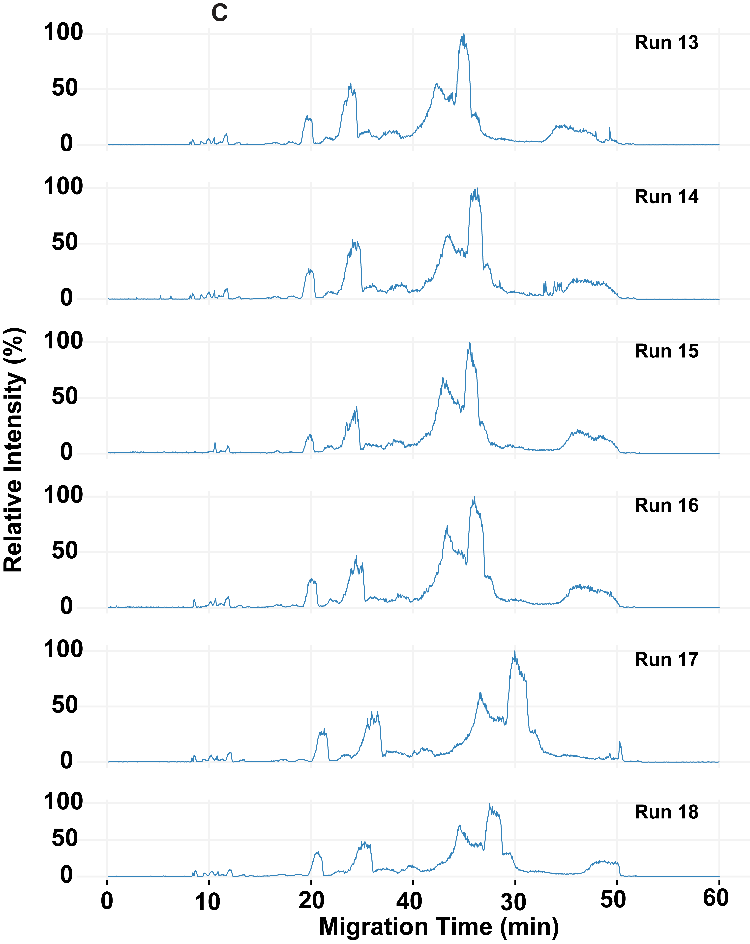

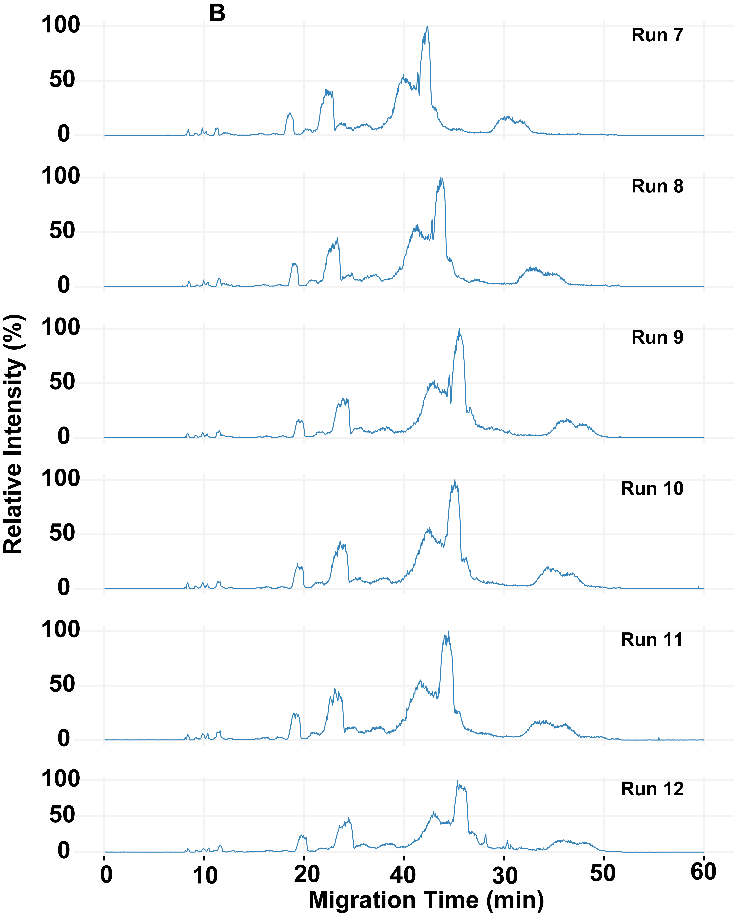


**Figure S1:** Electropherograms for all histone runs (A-C).


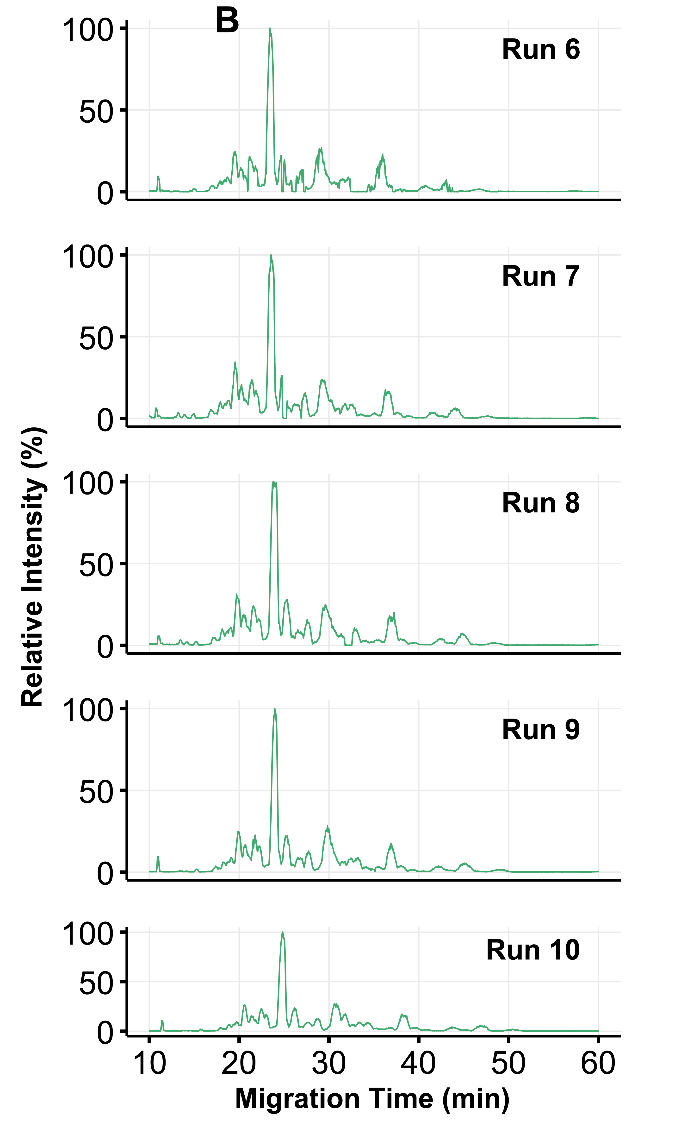

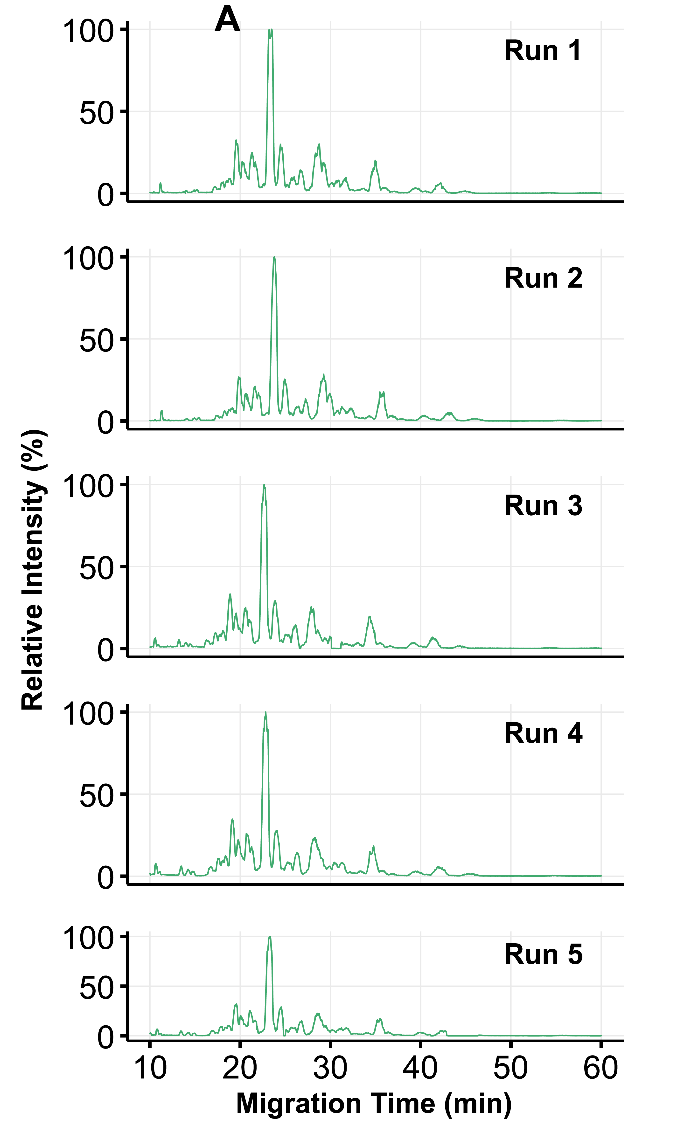


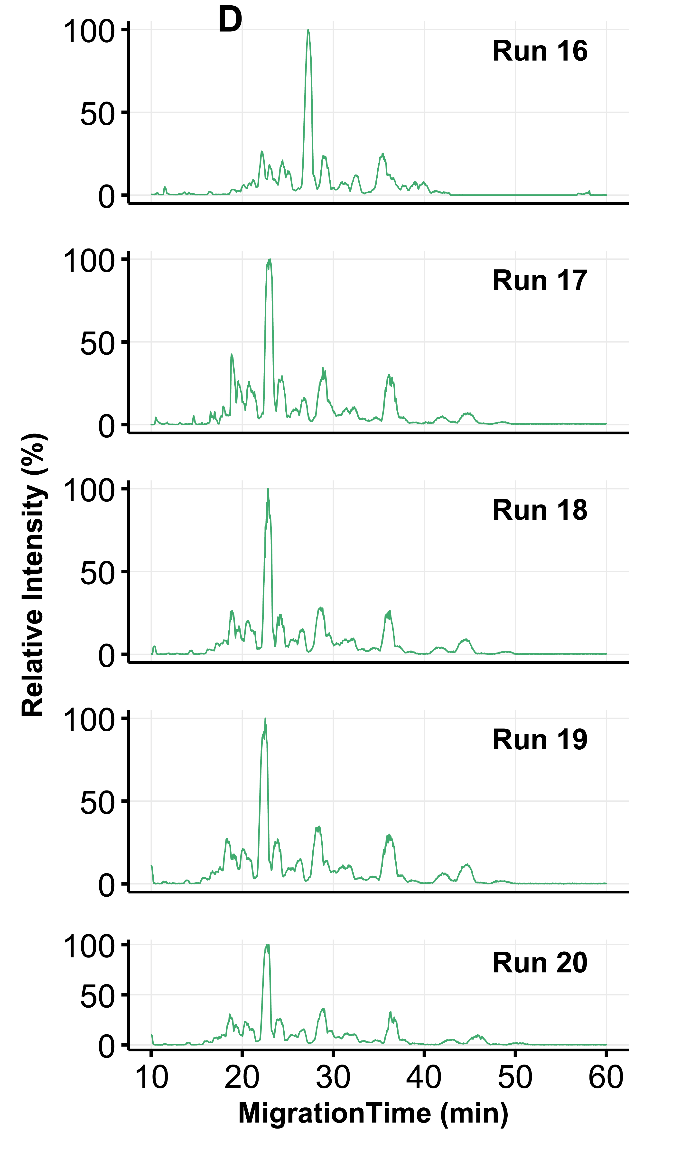

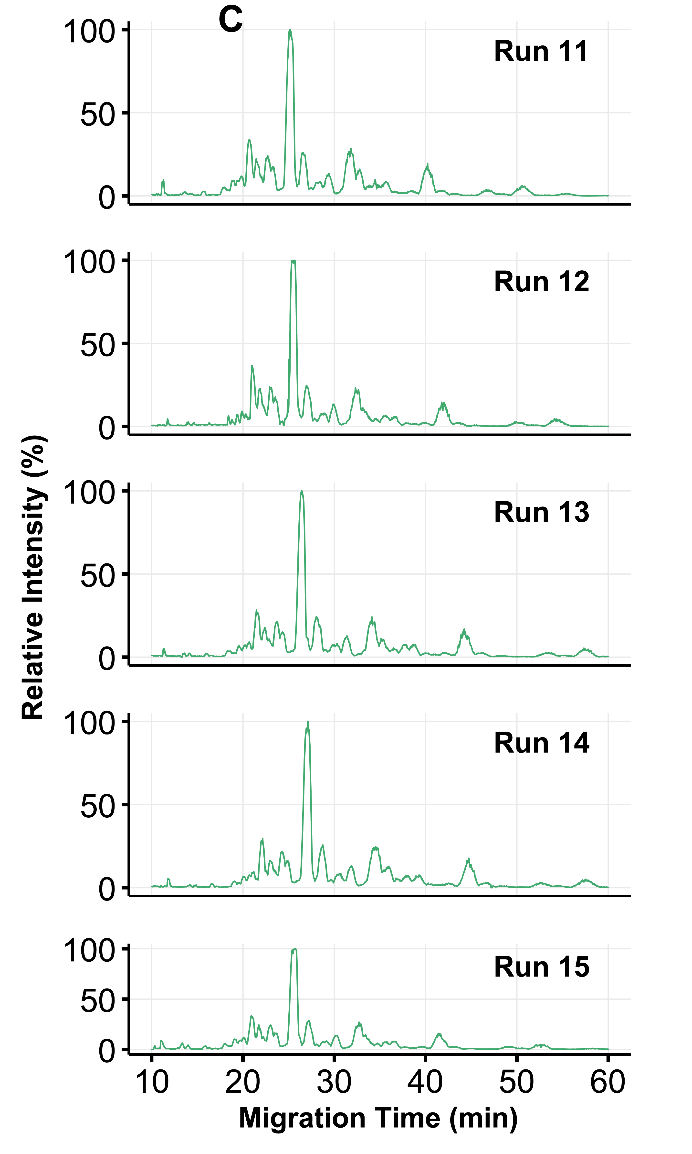


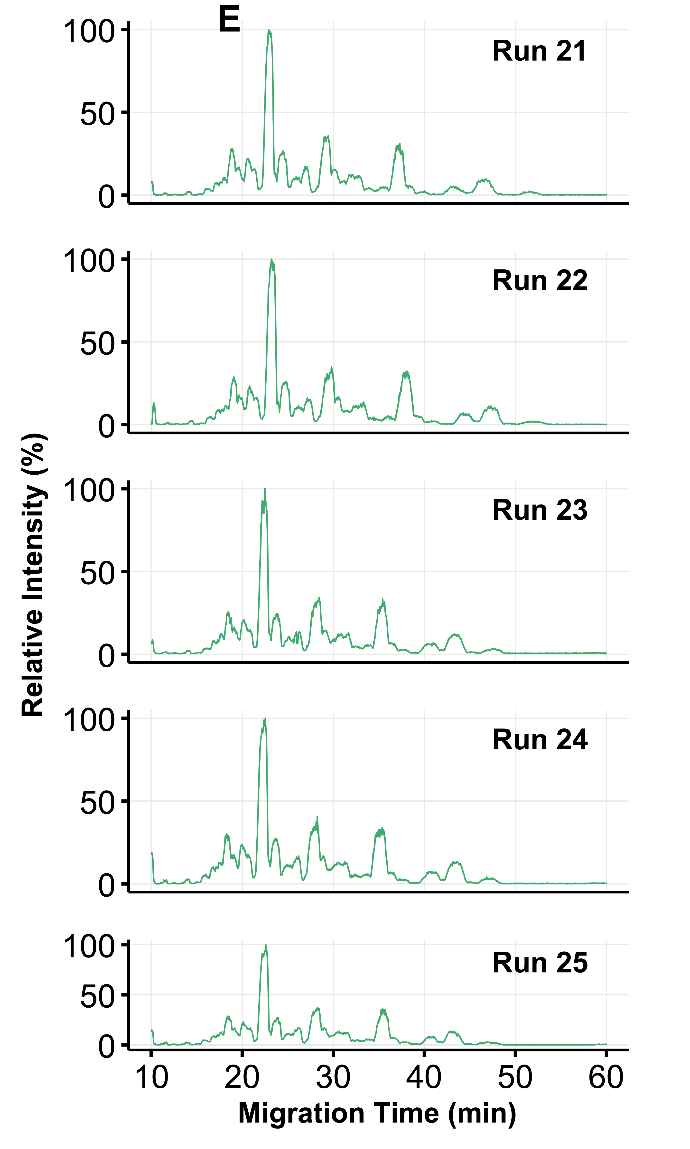

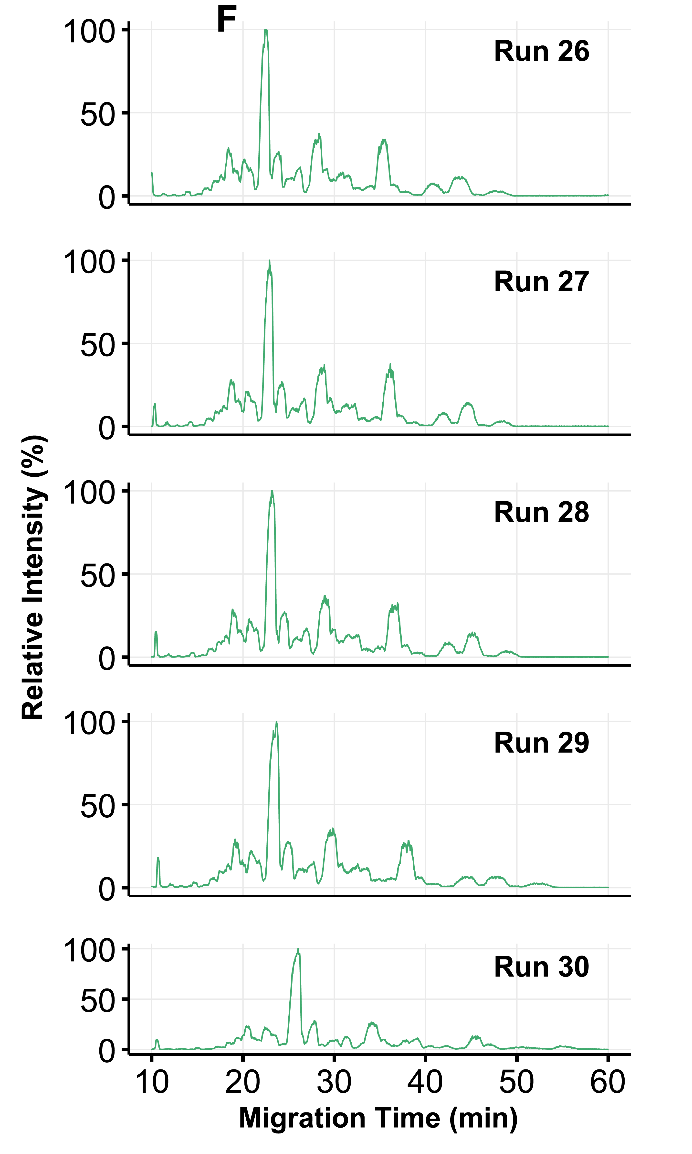


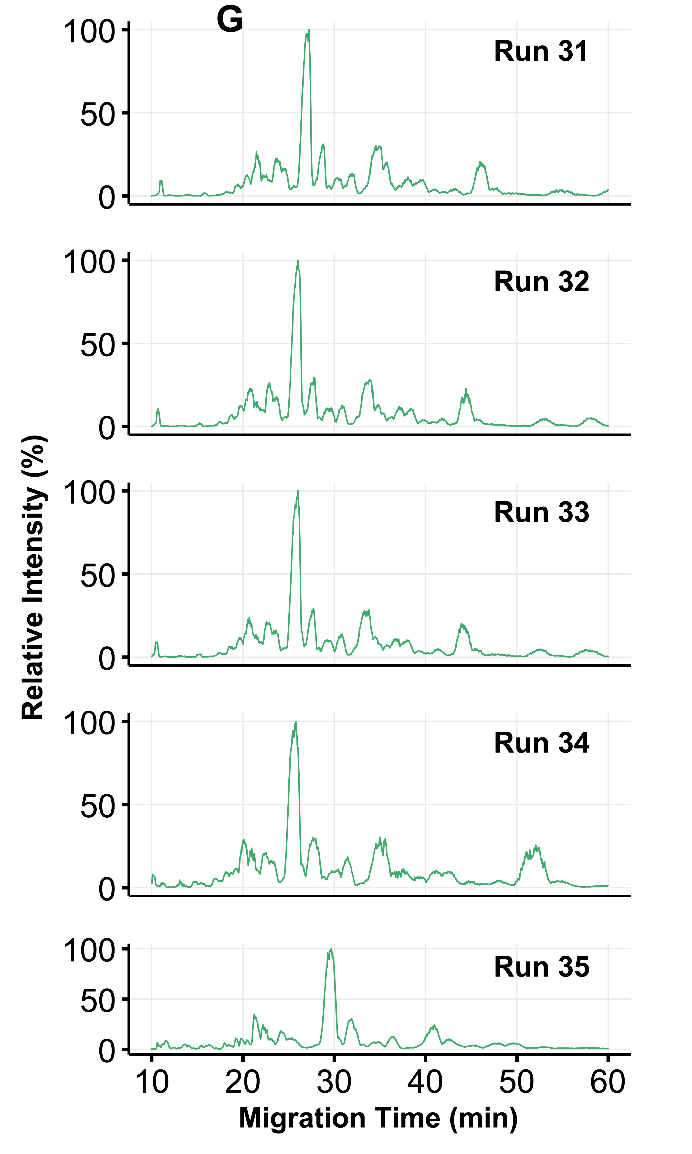


**Figure S2:** Electropherograms for all E. coli runs (A-G).
